# Xenogenic (porcine) acellular dermal matrix is useful for the wound healing of severely damaged extremities

**DOI:** 10.3892/etm.2014.1490

**Published:** 2014-01-20

**Authors:** ZHAOXIN ZHANG, LEI LV, MASUT MAMAT, ZHAO CHEN, LIHUA LIU, ZHIZHONG WANG

**Affiliations:** Department of Burn and Wound Repair Surgery, People’s Hospital of Xinjiang Uygur Autonomous Region, Urumqi, Xinjiang 830021, P.R. China

**Keywords:** xenogenic (porcine) acellular dermal matrix, damaged wounds, wound healing

## Abstract

This study was conducted to investigate the possibility of improving the success rate of patient treatment and promoting wound healing by utilizing xenogenic (porcine) acellular dermal matrix (XADM) to cover large areas of severely damaged wounds. Patients with severely damaged large-area wounds (56 cases) were enrolled in the study from May 2002 to May 2012. All patients admitted to hospital received a rapid infusion via intravenous access to maintain an effective circulating blood volume and to correct disorders of water and electrolytes. The wounds were exposed and covered with XADM during the initial surgery. All patients subsequently received secondary stage surgery. Of the patients, 47 cases received an autologous skin graft for wound closure, six cases underwent wound repair with a local flap and three cases underwent wound repair with an axial flap. There were two cases of amputation and three cases of mortality. The cases of two of the patients are described in detail. XADM was demonstrated to reduce the risk of emergency during surgery and improve the success rate of wound healing and patient treatment.

## Introduction

Avulsion refers to the violent separation of skin and soft tissue from muscle and bone tissue (1), and the wound closure of avulsion injuries is a complex surgical issue. The wound healing is highly challenging, particularly when accompanied by open wounds and vascular injury of the extremities. This is due to such injuries being associated with hypovolemic (or traumatic) shock, the exposure of important structures (nerves, tendons, blood vessels, etc.), serious contamination of wounds and severe combined wound injuries, as well as other factors. All the aforementioned factors may enhance the risk of first-line wound healing and thus cause failure of the surgery. Therefore, the thorough removal of devitalized tissue, fracture fixation and the appropriate use of blood tissue for covering the wound all form the basis of wound healing (2–4).

Xenogenic (porcine) acellular dermal matrix (XADM) is prepared by removing all the layers of the skin epidermis and all the cellular components of the dermis, while retaining the dermal collagen composition and basic structure, as well as the basement membrane components. The rejection of a skin graft is primarily caused by cell-mediated immunity. Therefore, such a matrix causes hardly any host rejection response and may induce normal tissue remodeling, on the condition that strong immunogenic cell components are removed from the matrix (5). An acellular biological covering demonstrates good air permeability, good adhesion to the wound and reduces the amount of exudate and bleeding, in addition to having a certain softness. In addition, such a covering is not easily broken and does not undergo significant water-loss or shrinking, which is quite convenient, particularly when the covering is applied to uneven or large bending wounds. The biofilm matrix forms a barrier that effectively isolates the wound, thus reducing the possibility of bacterial infection, providing a moist environment for the wound, promoting the migration of epithelial cells, inducing host fibroblast and endothelial cell proliferation, and promoting the ordered ingrowth of capillaries and rearrangement of the collagen fibers (6). Furthermore, the covering reduces the loss of moisture, calories, protein and electrolytes from the wound and reduces the elevated metabolic state, in addition to the systemic inflammatory response syndrome, thus stabilizing the environment of the whole body (7–9). XADM creates a local and suitable microenvironment for wound healing. In such a relatively confined, moist microenvironment with a suitable pH, necrotic tissues are enzymatically decomposed and the physiological structures are restored. In addition, such an environment promotes the rapid proliferation of fibroblasts and endothelial cells, the growth of granulation tissue and the vascularization of the wound to achieve optimum surgical results.

It has been demonstrated that XADM is able to effectively protect a wound when it is used in the treatment of deep burn wounds following excision (10). XADM also reduces the loss of water, proteins and electrolytes to prevent deep-degree expansion of the wound. In the present study, a first-stage surgical debridement of the wound and the application of XADM coverings, with secondary stage wound-closure surgery were performed for the reparation of extremities severely damaged by avulsion.

## Materials and methods

### General data of patients

A total of 56 patients with acute traumatic soft tissue damage were enrolled in the Burn Unit of the People’s Hospital of Xinjiang Uygur Autonomous Region for wound-repair surgery (Urumqi, China) from May 2002 to May 2012. Of the patients, 46 cases were male and 10 were female and they were aged 26–42 years, with a mean age of 34.27±1.28 years. The time from injury to hospital admission ranged from 4 h to 10 days. All patients exhibited varying degrees of hypovolemic shock or symptoms of septic shock (Table I). Based upon the classification criteria of avulsion from Arnez *et al* (1), these patients all had injuries of circumferential multi-plane degloving, which are classified as fourth-degree avulsions. The study was approved by the ethics committee of The People’s Hospital of Xinjiang Uygur Autonomous Region. Prior written and informed consent was obtained from each patient.

### Surgery

Prior to receiving any other treatments following injury, the patients underwent infusion via intravenous access following admission. A mixture of crystalloid and colloid was infused. The wound was bandaged to prevent bleeding. The contaminated, significantly necrotic tissues and ischemic tissues were cleared away during the surgery, while physiological structures affected by partial necrosis and vital vessels, nerves and tendon tissues were retained as much as possible as these may be used as stents during subsequent reconstructive surgery. The wounds were covered with XADM, which was purchased from JiangSu Unitrump Bio-medical Technology Co., Ltd. (Nantong, China). A suction drainage tube was placed subcutaneously and the sterile covering was gently placed and bandaged. The entire surgery time was limited to 1–2 h. At 5–7 days after the first stage surgery, the secondary stage surgery with thick flaps or an autologous skin graft for wound closure was performed.

## Results

Of the 56 patients, 47 cases who received an autologous skin graft had their wounds repaired successfully. A further six cases were treated with local flaps and three cases were treated with axial flaps for wound repair. Of all the patients, two cases underwent amputation due to irreparable damage and three cases passed away due to acute renal failure and hemorrhagic shock.

### Case 1

**Figure 1:**
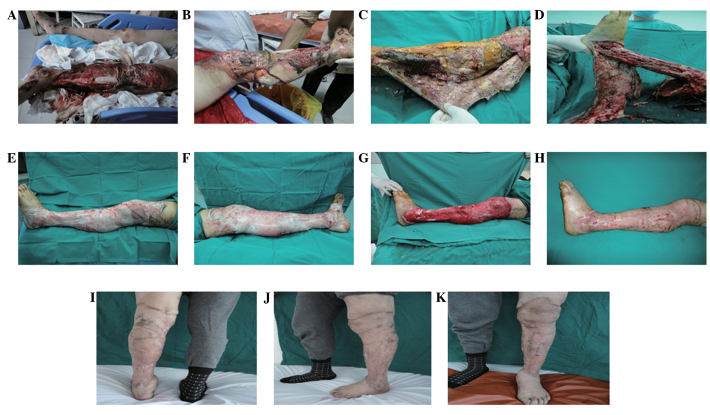
Case 1: Male, aged 19, admitted to hospital 10 days after a car accident trauma. Diagnosis: i) Avulsion and severe infection of skin and soft tissues of the left lower extremity; ii) fracture of the left tibia and fibula; and iii) septic shock. (A and B) A wide range of soft tissue loss and wound contamination between the knee and ankle of the left leg was observed. In addition, a large area of necrotic tissue and heavy purulent discharge were identified and the tibia and knee joint cavity were partially exposed. (C) A number of muscles of the left leg were noted to be affected by infection and necrosis during the surgery. A number of areas of sediment and purulent discharge were observed on the wound. (D) The muscles were automatically self-separated from the gastrocnemius and soleus and tibia and fibula after the surface of the skin was cut. (E and F) Following debridement, the ruptured Achilles tendon was connected with the triceps tendon. The skin and subcutaneous tissue affected by infection and necrosis were removed and a large sheet of XADM was used to cover the wound. Several holes were created in the covering for drainage (on the lateral and medial left lower extremity). (G) Following 10 days of XADM covering, the wound granulation tissue appeared fresh with fine particles and wound bed preparation was complete. (H) Wound closure was achieved 12 days after the conduction of a large sheet edge autologous skin graft of the left lower extremity. (I–K) The follow-up was conducted one year following the surgery and the shape and function of the left lower extremity exhibited good recovery. XADM, xenograft (porcine) acellular dermal matrix.

A 19 year-old male was admitted to hospital 10 days after a car accident trauma. The admission examination results were as follows: Temperature, 39.8°C; heart rate, 124 beats/min; respiration, 24 beats/min; and blood pressure, 75/50 mmHg. The results of the laboratory examination were as follows: white blood cell count, 3.5×10^9^/l. An X-ray of the left lower extremity indicated left tibia and fibula fractures. The diagnosis concluded there was: i) Avulsion and severe infection of the skin and soft tissues of the left lower extremity; ii) fracture of the left tibia and fibula; and iii) septic shock. Visual examination (Fig. 1) revealed a wide range of soft tissue loss and wound contamination below the knee, to the ankle of the left leg. In addition, it indicated a large area of necrotic tissue and heavy purulent discharge, and the tibia and knee joint cavities were partially exposed (Fig. 1A and B). Certain muscles of the left leg were noted to be affected by infection and necrosis during the surgery. A number of areas of sediment and purulent discharge were observed on the wound (Fig. 1C). The muscles had automatically self-separated from the gastrocnemius and soleus and the tibia and fibula after the surface of the skin was cut (Fig. 1D). Following debridement, the ruptured Achilles tendon was connected with the gastrocnemius and soleus (Fig. 1E and F). The sections of the skin and subcutaneous tissue affected by infection and necrosis were removed and a large sheet of XADM was used to cover the wound. Several holes were opened in the covering for drainage on the left lateral leg (Fig. 1E) and left medial leg (Fig. 1F). Following 10 days of XADM covering, the wound granulation tissue appeared fresh with fine particles and the wound bed preparation was complete (Fig. 1G). Wound closure was achieved 12 days after the conduction of a large sheet edge autologous skin graft of the left lower extremity (Fig. 1H). The follow-up was conducted one year after the surgery and the shape and function of the left lower extremity exhibited good recovery (Fig. 1I–K).

### Case 2

**Figure 2:**
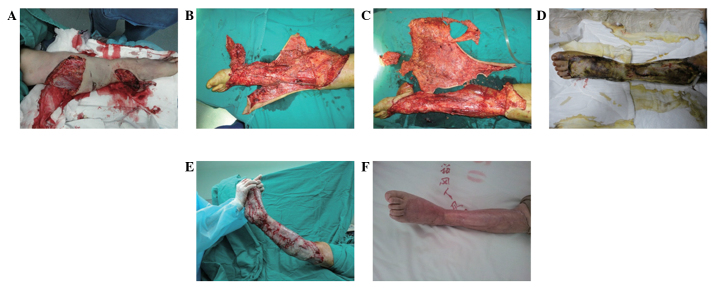
Case 2: Female, aged 45, admitted to hospital 4 h after a motor vehicle accident. Diagnosis: i) Avulsion of the left lower extremity; ii) fracture of the left tibia; and iii) hemorrhagic shock. (A and B) The skin and subcutaneous soft tissue below the left knee were stripped off and separated from the deep fascia. (C) The wound bed tissue became swollen following debridement. Furthermore, physiological structures and the normal tissues were mixed. Therefore, the success rate of skin grafting was likely to be relatively low. (D) A large sheet of XADM was used to cover the wound and several holes were opened in it for drainage. (E) The wound bed preparation was completed following 10 days of debridement. An autologous skin graft was subsequently performed for wound closure. (F) The follow-up was conducted one year following the surgery. Good recovery of the shape and function of the limb was exhibited. XADM, xenograft (porcine) acellular dermal matrix.

A 45 year-old female was admitted to hospital 4 h after a motor vehicle accident. The diagnosis was as follows: i) Avulsion of the left lower extremity; ii) fracture of the left tibia; and iii) hemorrhagic shock. As presented in Fig. 2, the skin and subcutaneous soft tissue below the left knee were stripped off and separated from the deep fascia (Fig. 2A and B). The wound bed tissue became swollen following debridement. Furthermore, the physiological structures and normal tissues were mixed. Therefore, the success rate of a skin graft was likely to be relatively low (Fig. 2C). A large sheet of XADM was used to cover the wound and several holes were opened in it for drainage (Fig. 2D and E). The wound bed preparation was complete following 10 days of debridement and an autologous skin graft was subsequently performed for wound closure (Fig. 2F). The follow-up was conducted one year after surgery. The shape and function of the left lower extremity demonstrated good recovery (Fig. 2G). These results indicate that the XADM is useful in the wound healing of severely damaged extremities.

## Discussion

Gangrene, infection of avulsed tissues and even certain serious complications such as systemic sepsis are likely to occur if damaged wounds are not treated in a timely and effective manner. Therefore, early-stage thorough debridement, drainage and conservation of physiological structures are important in the repair of avulsion injuries. In the present study, it was demonstrated that XADM reduced the risk of emergency during surgery and had good success rates for wound healing and patient treatment. The use of XADM in the application of severely damaged wound repair, to the best of our knowledge, has not been previously reported.

In comparison with certain traditional techniques, such as the Integra^®^ artificial dermis (11–13) and closed suction drainage techniques (14–16), the cost of XADM is low. XADM is easily prepared from abundant resources. Furthermore, the side-effects caused by XADM are usually less than those of the Integra artificial dermis and the closed suction drainage techniques. These advantages suggest that XADM may be a good type of material for the treatment of severely damaged wounds.

## Figures and Tables

**Table I:** Data of the patients (n=56).

Symptoms	Number of cases
Shock	
Moderate or severe hypovolemic	39
Early	17
Fractures	
Open	17
Closed	12
Traumatic brain injury	8
Thoraco-abdominal injury	
Liver and spleen rupture	5
Pulmonary contusion	2
Intestinal perforation	2
Diaphragmatic hernia	1
Urinary tract trauma	
Kidney laceration	2
Urethral rupture	2
Bladder injury	1
Injuries	
Unilateral extremity	42
Two or more physical injuries	14
